# Enhanced Platelet Activation Mediates the Accelerated Angiogenic Switch in Mice Lacking Histidine-Rich Glycoprotein

**DOI:** 10.1371/journal.pone.0014526

**Published:** 2011-01-17

**Authors:** Maria Ringvall, Åsa Thulin, Lei Zhang, Jessica Cedervall, Nobuko Tsuchida-Straeten, Willi Jahnen-Dechent, Agneta Siegbahn, Anna-Karin Olsson

**Affiliations:** 1 Department of Medical Biochemistry and Microbiology, Uppsala Biomedical Center, Uppsala University, Uppsala, Sweden; 2 Biointerface Laboratory, Department of Biomedical Engineering, RWTH Aachen University, Aachen, Germany; 3 Department of Medical Sciences, Uppsala University Hospital, Uppsala University, Uppsala, Sweden; Emory University, United States of America

## Abstract

**Background:**

The heparin-binding plasma protein histidine-rich glycoprotein (HRG; alternatively, HRGP/HPRG) can suppress tumor angiogenesis and growth *in vitro* and *in vivo*. Mice lacking the HRG gene are viable and fertile, but have an enhanced coagulation resulting in decreased bleeding times. In addition, the angiogenic switch is significantly enhanced in HRG-deficient mice.

**Methodology/Principal Findings:**

To address whether HRG deficiency affects tumor development, we have crossed HRG knockout mice with the RIP1-Tag2 mouse, a well established orthotopic model of multistage carcinogenesis. RIP1-Tag2 HRG^−/−^ mice display significantly larger tumor volume compared to their RIP1-Tag2 HRG^+/+^ littermates, supporting a role for HRG as an endogenous regulator of tumor growth. In the present study we also demonstrate that platelet activation is increased in mice lacking HRG. To address whether this elevated platelet activation contributes to the increased pathological angiogenesis in HRG-deficient mice, they were rendered thrombocytopenic before the onset of the angiogenic switch by injection of the anti-platelet antibody GP1bα. Interestingly, this treatment suppressed the increase in angiogenic neoplasias seen in HRG knockout mice. However, if GP1bα treatment was initiated at a later stage, after the onset of the angiogenic switch, no suppression of tumor growth was detected in HRG-deficient mice.

**Conclusions:**

Our data show that increased platelet activation mediates the accelerated angiogenic switch in HRG-deficient mice. Moreover, we conclude that platelets play a crucial role in the early stages of tumor development but are of less significance for tumor growth once angiogenesis has been initiated.

## Introduction

Histidine-rich glycoprotein (HRG; alternatively, HRGP/HPRG) has been identified as an angiogenesis inhibitor *in vitro* and *in vivo* by us and others [1], [2], [3]. HRG is a 75 kDa single chain heparin-binding plasma protein produced by the liver [4]. Structurally, HRG consists of three distinct domains; an amino-terminal part with two cystatin (cysteine proteinase inhibitor)-like domains, which classifies HRG as a member of the cystatin superfamily together with e.g. kininogen and fetuin, a central histidine/proline-rich (His/Pro-rich) domain organized in tandem repeats of a consensus GHHPH motif, and a carboxy-terminal domain. Multiple binding partners for HRG have been reported, such as heparin/heparan sulfate, divalent cations, components in the coagulation-fibrinolysis system; plasminogen and fibrinogen, as well as components in the immune system such as T lymphocytes, monocytes/macrophages and immunoglobulins [4]. Monocytes were previously believed to express HRG, since HRG binds to the cell surface of monocytes, but more recent data demonstrate that RNA is found only in the liver [5]. In addition, HRG has been reported to be present within platelets and megakaryocytes [6]. Mice lacking the HRG gene are viable and fertile, but have an enhanced coagulation resulting in decreased bleeding times [7]. HRG thereby exemplifies one of several molecules regulating both angiogenesis and hemostasis [8].

Platelets are anuclear cellular fragments derived from megakaryocytes in the bone marrow and play a crucial role in regulating blood hemostasis. At sites of blood vessel injury, platelets are activated and aggregate at the site of the damaged endothelium to prevent hemorrhage. Besides their role in hemostasis, platelets contribute to non-hemostatic processes such as immunity, tumor metastasis and angiogenesis [9], [10], [11]. Platelets contain a large number of both pro- and antiangiogenic factors and regulation of angiogenesis by platelets was suggested as early as 1968 [12]. Examples of positive regulators of angiogenesis found in platelets are VEGF-A, VEGF-C, platelet-derived growth factor (PDGF) and fibroblast growth factor-2 (FGF-2), while negative regulators include thrombospondin, platelet factor-4 (PF-4) and plasminogen activator inhibitor type-1 (PAI-1) [8], [13]. Despite their content of both positive and negative regulators of blood vessel formation, platelets have in several different experimental settings been shown to stimulate angiogenesis [11], [14], [15], [16], [17]. It is well known that cancer patients have an increased turnover of platelets and increased risk of thrombotic occlusion, as a result of increased platelet activation. Both increased coagulation and platelet activation have been demonstrated to stimulate tumor angiogenesis, as well as metastasis, and can therefore contribute to disease progression [11], [18].

In the present study we address how lack of HRG affects tumor growth. For this purpose we have crossed HRG-deficient mice with the orthotopic RIP1-Tag2 mouse model of insulinoma [19]. These mice carry the SV40 T-antigens under the control of the insulin promoter, which is expressed in the islets of Langerhans in the pancreas. The Rip1-Tag2 is an orthotopic model of multistage carcinogenesis, believed to better reflect the stepwise process of tumor development via distinctive stages, than conventional subcutaneous models with injected tumor cells. One of these stages represents the “angiogenic switch”. Moreover, we investigate whether the deregulated hemostasis in HRG-deficient mice contribute to the elevated angiogenic switch, previously reported in these mice.

## Materials and Methods

### Antibodies

The following primary antibodies were used in this study: anti-Ki67 (Dakocytomation/ M7249), anti-cleaved caspase-3 (Cell Signalling/9661), anti-CD31 (BD/557355), anti-CD41 (BD/553847) and anti- GP1bα (Emfret/R300). The following direct-conjugated antibodies were used for flow cytometry: PE-conjugated anti-mouse GPIIb/IIIa (JON/A/M023-2/Emfret), PE-conjugated anti-P-selectin (Emfret/M130-2) and FITC-conjugated anti-GPIX (Emfret/M051-0). Control antibodies for flow cytometric analysis were: PE-conjugated rat IgG (Emfret/P190-2), FITC-conjugated rat IgG (Emfret/P190-1), rabbit IgG (Cedarlane/CLRB00) and FITC-conjugated rat IgM (555583/BD). The following secondary antibodies were used: anti-rat Alexa488 (Molecular Probes/A21208), anti-rabbit (BA-1000/Vector Laboratories), anti-mouse (BA-9200/Vector Laboratories) and anti-rat (BA-9400/Vector Laboratories).

### Ethics Statement

All animals were handled in strict accordance with good animal practice as defined by the relevant national and/or local animal welfare bodies, and all animal work was approved by the Uppsala University board of animal experimentation (C279/7) and thus performed according to the United Kingdom Coordinating Committee on Cancer Research (UKCCCR) guidelines for the welfare of animals in experimental neoplasia [20].

### RIP1-Tag2 HRG^+/+^ and RIP1-Tag2 HRG^−/−^ mice

All mouse strains were on a pure C57BL/6 genetic background. HRG deficient female mice were mated with RIP1-Tag2 positive (RT2) males to produce founder mice for breeding of RT2/HRG^−/−^ and RT2/HRG^+/+^ littermates. From 10 weeks of age, all RIP1-Tag2 positive mice received drinking water supplied with 5% sucrose to relieve hypoglycemia induced by the insulin-secreting tumors. DNA extracted from tail biopsies was used as the template for genotyping by PCR. The following primers were used: Forward HRG primer 5′-CCTGGGGTCAAAGTGAACATGC-3′; reverse HRG wild-type primer 5′-CGCTCTGTCCAAGTGGGCGTCA-3′; reverse knockout HRG primer (located in neomycin cassette) 5′-TTGTGTAGCGCAAGTGCCAGCG-3′; forward Tag2 primer 5′-GGACAACCACAACTAGAATGCAG-3′; reverse Tag2 primer 5′-CAGAGCAGAATTGTGGAGTGG-3′.

### Dissection and quantification of angiogenic islets and tumors

Twelve or 15 weeks old RT2/HRG^+/+^ or RT2/HRG^−/−^ mice were anesthetized by intraperitoneal injection of 2% avertin. Heart perfusion was done with 10 ml of phosphate buffered saline (PBS) (pH 7.4) followed by 10 ml of 2% paraformaldehyde (PFA) in PBS (pH 7.4). Pancreases were removed from the abdominal cavity and tumors and angiogenic islets were dissected away from exocrine pancreas under a stereo dissection microscope at ×10 magnification. Tumors and angiogenic islets (defined as blood containing hyperplastic islet with a diameter of <1 mm) were measured and counted. Tumor volumes were calculated by the formula ((π/6)×width^2^×length). Tumors and angiogenic islets were stored in a 30% sucrose in PBS solution over night at 4°C. The material was frozen in Tissue-Tek® O.C.T. and stored at −70°C until further processing.

### Immunohistochemistry

Frozen sections of mouse tissue were fixed in ice-cold methanol, washed in PBS, incubated with 1% H_2_O_2_ to quench endogenous peroxidases and blocked in 3% bovine serum albumin (BSA)+20% horse serum in PBS for 30 min. To reduce background signal from endogenous biotin in the pancreas, an avidin/biotin blocking kit (SP-2001/Vector Laboratories) was applied before addition of antibodies. Sections were incubated with primary antibody for 2 hours at room temperature or at 4°C over night. Primary antibodies were diluted in blocking buffer as follows: CD31 1∶500, cleaved caspase-3 1∶200, Ki67 1∶100 and CD41 1∶300. For enzymatic detection, sections were incubated with 1% H_2_O_2_ prior to blocking. In order to detect primary antibody binding sites, sections were incubated with biotinylated anti-rat (CD31, Ki67, CD41) or anti-rabbit (cleaved caspase-3) antibody for 30 minutes diluted 1∶300 in blocking buffer. After washing, sections were incubated with HRP-conjugated streptavidin (SA-5004/Vector Laboratories) diluted 1∶200 in blocking buffer for 30 minutes at room temperature. Binding sites were subsequently visualized with a HRP substrate using the AEC Peroxidase Substrate Kit (SK-4200/Vector Laboratories). Hematoxylin staining was used to visualize nuclei.

### Quantification of proliferation, apoptosis, vascularization and platelets in tissue

The proportion of proliferative and apoptotic cells in tumors was assessed by manual counting of the total number of cells and the number of Ki67 and cleaved caspase-3 positive cells respectively. A total number of 5000 cells were counted for Ki67 and approximately 30 000 cells were counted for cleaved caspase-3 in each group, in tumor tissue derived from n = 3 individuals/group (cleaved caspase-3) or n = 4 individuals /group (Ki67).

Tumor vascularization (length, volume and surface density) was quantified by stereology as previously described in tissue from n = 7 individuals/group [21].

Analysis of CD41 positive platelets in kidney from healthy mice were done by manual counting of all positively stained platelets in sections from whole kidney from n = 5–6 individuals/group. The data is given as average number of platelets/field. The proportion of platelets in tumor tissue is much higher compared to healthy tissue and the area stained positive for CD41 in tumors was therefore quantified using the image analysis program Image J 1.42 software (National Institutes of Health). The data is presented as % of total tumor area.

### Blood sampling

Blood was drawn by cardiac puncture from isoflurane anesthetized mice and anti-coagulated with 0,00129 M citrate.

### Platelet aggregation

Citrated blood was centrifuged at 1800 rpm for 5 minutes and the supernatant was further centrifuged at 800 rpm for 6 minutes to obtain platelet-rich plasma (PRP). To obtain platelet-poor plasma (PPP), the supernatant was additionally centrifuged at 3000 g for 10 minutes. Platelet count was adjusted to 350×10^3^ platelets/µl with PPP or Tyrode's buffer. Aggregation was induced with 20 µg/ml collagen, 0,15 U/ml bovine thrombin or 20 µM ADP. Platelet aggregation activity was measured in a platelet aggregometer (Labor FIBRINTIMER®, Ahrensburg, Germany) where light transmission is recorded over time. Light transmission is increased as platelets aggregate.

### Platelet analysis in mouse blood with flow cytometry and cell counting

For flow cytometry, 5 µl citrated blood was incubated with antibody and HEPES-buffered saline (145 mM NaCl, 5 mM KCl, 1 mM MgSO_4_, 10 mM HEPES, pH 7,4) in a total volume of 50 µl. Dilution of antibodies were 1∶10 for anti-fibrinogen, anti-GPIIb/IIIa, anti-GPIX and anti-P-selectin. ADP activation was done with 5–20 µM ADP for 15 minutes at room temperature and stopped by addition of 400 µl 0,4% PFA/PBS (pH 7,4) and incubation at room temperature for 15 minutes. Thrombin activation was performed using 0,01 U/ml thrombin (HCT-0020 Haematologic technologies inc), together with 1 mM Pefabloc^FG^ (Pentapharm) to prevent fibrin clot formation. Activation was ended at different time points by fixation of cells with 400 µl 0,4% PFA/PBS (pH 7,4). Staining with antibodies was done after fixation and washing of thrombin activated samples. Samples were washed with 1 ml PBS and centrifuged at 1500g for 15 minutes and cells resuspended in 0,5 ml PBS prior to analysis. Flow cytometric analysis was performed and platelets were gated to see mean fluorescence intensity (MFI) of the respective antibody staining for the platelet population. For platelet numbers, either whole blood was stained as described above and GPIX positive cells given as percentage of the total number of cells in whole blood, or the absolute number of platelets/volume in whole blood was determined using a cell counter (Coulter).

### Blocking of platelet activation by Plavix

C57BL/6 female mice, wild type (Taconic, Denmark) and HRG^−/−^, were treated with Plavix (clopidogrel; Sanofi pharma) at 0,25 mg/ml (30 mg/kg/day) or 0,50 mg/ml (60 mg/kg/day) in their drinking water during three days. Control mice received only water. Mice were anesthetized with isoflurane, blood drawn by cardiac puncture and anti-coagulated with 0,00129 M citrate before analysis of ADP-induced activation of the fibrinogen receptor and the relative number of GPIX positive platelets in whole blood.

### GP1bα treatment of RT2/HRG^+/+^ and RT2/HRG^−/−^ mice

*Angiogenic islets:* GP1bα treatment of RT2/HRG^+/+^ or RT2/HRG^−/−^ female mice started with 4 µg GP1bα antibody/g body weight (60 µg/mouse) at five weeks of age (day 0), administered by intra-peritoneal injection. The treatment was continued with 2 µg GP1bα antibody/g body weight (30 µg/mouse) every third day until day 9. The mice were then allowed to recover platelet levels for five days prior pancreatic dissection and counting of angiogenic islets at day 14, when the mice were seven weeks old.

*Tumor volume:* GP1bα treatment of RT2/HRG^+/+^ or RT2/HRG^−/−^ female mice started at nine weeks of age (day 0) and continued with injections every third day until day 12. The mice were then allowed to recover platelet levels for nine days prior pancreatic dissection and analysis of tumor volume at day 21, when the mice were 12 weeks old.

### Statistical analysis

All statistical analyses in this study were performed using the non-parametric two-tailed Mann-Whitney test. The Mann-Whitney test should be used instead of the parametric Student's t-test when the number of observations in each group are few (approximately <50). * is defined as p≤0.05, ** as p≤0.01 and *** as p≤0.001.

## Results

### Generation of HRG-deficient RIP1-Tag2 mice

With the aim to investigate how lack of HRG affects tumor angiogenesis and growth *in vivo*, we crossed HRG-deficient mice with the RIP1-Tag2 model of spontaneous insulinoma [19]. This transgenic tumor model carries the SV40 T antigens under control of the rat insulin promoter, which is expressed in the islets of Langerhans in the pancreas. The RIP1-Tag2 mouse is an orthotopic model of multistage carcinogenesis and is considered to be a more accurate model for the stepwise development of tumors than conventional subcutaneous models with injected tumor cells. One of these stages represents the “angiogenic switch”, which is required for the transition from hyperplasia to neoplasia and further tumor growth. During this stage, the islet capillaries that are normally quiescent, are characterized by endothelial proliferation, vascular dilation and microhemorrhaging. Around 10% of the 400 islets present in a mouse pancreas adapt an angiogenic phenotype and out of these angiogenic islets 12–25% further develop into larger tumors [19].

**Figure 1:**
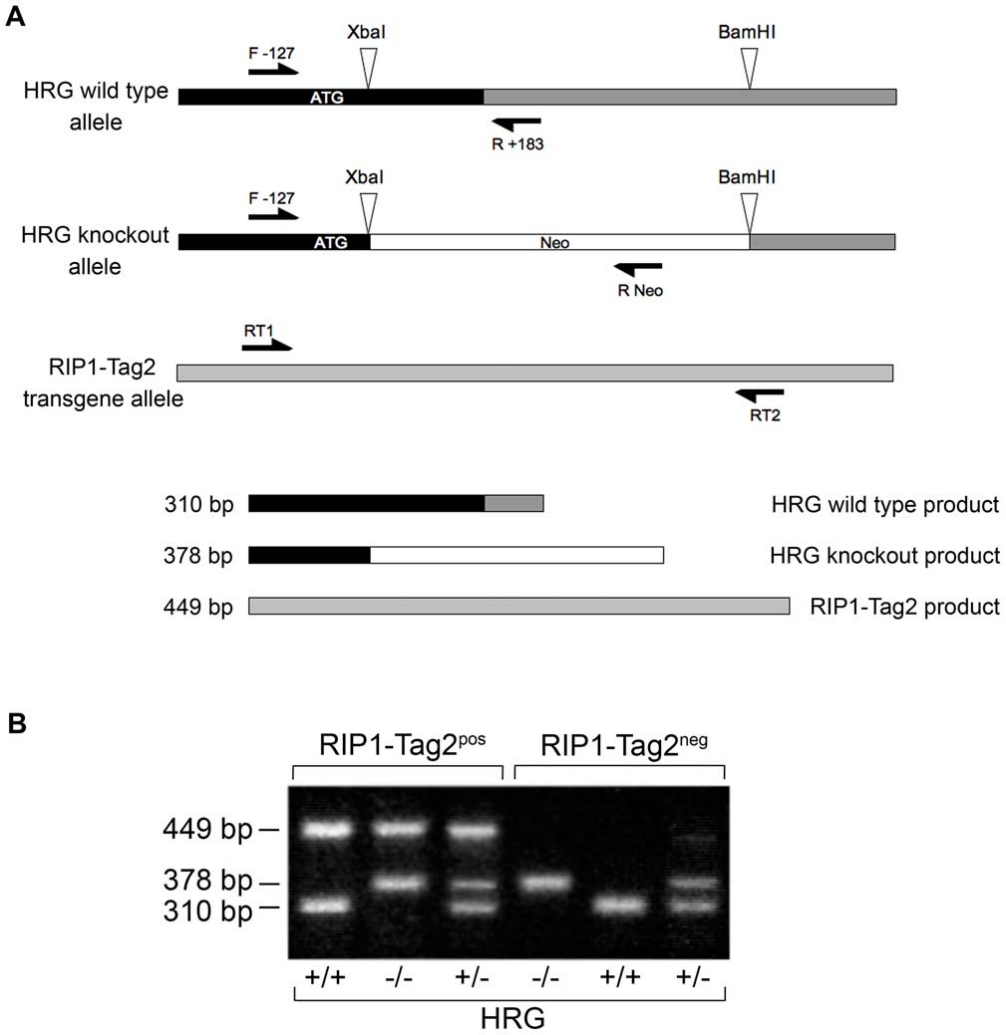
Genotyping PCR. Offspring from mating between RT2/HRG^+/−^ males and HRG^+/−^ females were genotyped by a five-primer PCR. (A) The five primers give three distinct PCR products. Primer pair F-127/R+183 gives a 310 bp HRG wild type allele product, primer pair F-127/RNeo gives a 378 bp HRG knockout allele product and primer pair RT1/RT2 gives a 449 bp RT2 transgene allele product. The location of each primer is indicated in the map by an arrow. (B) PCR products separated on a 2% agarose gel. All six possible genotypes are presented.

RIP1-Tag2 positive (RT2) HRG heterozygote (HRG^+/−^) males were mated with RIP1-Tag2 negative HRG^+/−^ females to enable analysis of HRG wild type (HRG^+/+^) and HRG knockout (HRG^−/−^) littermates in a RT2 positive genetic background. The genotypes of the mice were determined by PCR (Fig. 1A). The following PCR-products were generated; RT2 449 bp, HRG^+/+^ 310 bp, HRG^−/−^ 378 bp and HRG^+/−^ 310 and 378 bp (Fig. 1B).

### Accelerated tumor growth in HRG-deficient RIP1-Tag2 mice

**Figure 2:**
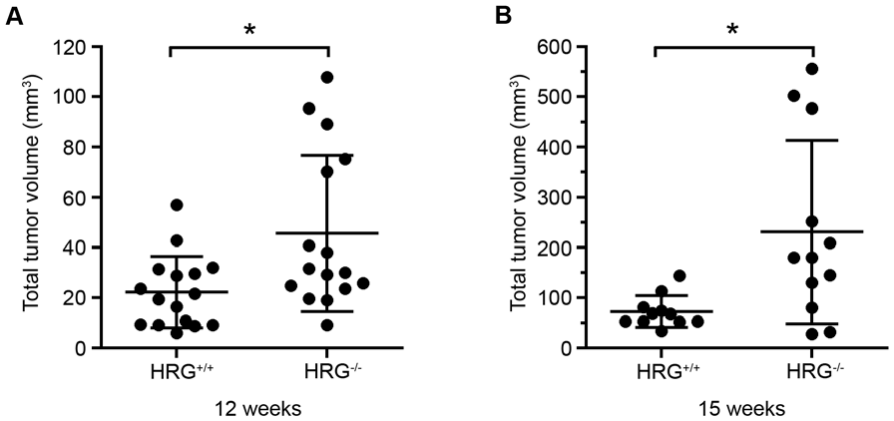
Tumor volume is increased in RT2/HRG^−/−^ compared to RT2/HRG^+/+^ mice. Tumors were dissected and measured. Tumor volumes were calculated by the formula ((π/6)×width^2^×length). Each dot represents the summarized tumor volume in mm^3^ from one mouse at (A) 12 weeks of age (n^+/+^ = 16; n^−/−^ = 16) and at (B) 15 weeks of age (n^+/+^ = 11; n^−/−^ = 12). Statistical analyses were performed with a two-tailed Mann-Whitney test, vertical bars represent standard deviation, * p≤0.05.

Analysis of tumor growth in RT2/HRG^+/+^ and RT2/HRG^−/−^ mice was performed on gender-matched groups at two ages, 12 and 15 weeks. The total tumor burden was assessed by two parameters; total tumor volume and number of tumors. At 12 weeks of age, the mean total tumor volume in the HRG^−/−^ group was two times higher than in the HRG^+/+^ group (Fig. 2A, table 1). Three weeks later, at 15 weeks of age, the difference between the two groups was further enhanced. At this time point the mean total tumor volume in the HRG^−/−^ group was more than three times higher than in the HRG^+/+^ group (Fig. 2B, table 1). The number of tumors did not differ between the groups at any age (data not shown).

**Table 1:** Mean tumor volume in RT2/HRG^+/+^ and RT2/HRG^−/−^ mice at 12 and 15 weeks.

	Mean total tumor volume ± SD (mm^3^)	
	12 weeks	15 weeks
RT2/HRG^+/+^	22,35±14,2	72,74±31,38
RT2/HRG^−/−^	45,69±31,11	231,4±182,5
Ratio (RT2/HRG^−/−^) vs. (RT2/HRG^+/+^)	2,04	3,18

### Tumors in HRG-deficient RIP1-Tag2 mice display increased proliferation

**Figure 3:**
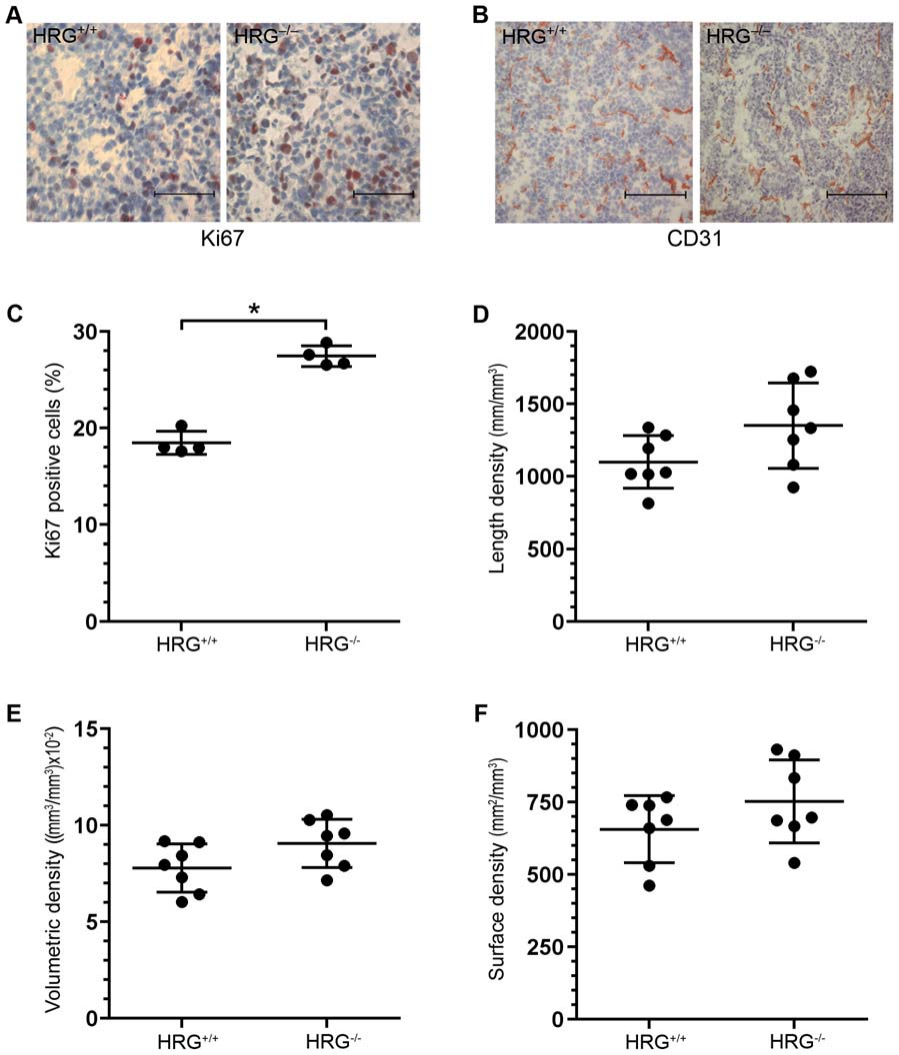
Proliferation and vascularization in tumors from RT2/HRG^+/+^ and RT2/HRG^−/−^ mice. (A, B) Immunohistochemical staining of tumor tissue from HRG^+/+^ and HRG^−/−^ mice directed against Ki67 (A) and CD31 (B). (C) Quantification of immunohistochemical staining directed against Ki67 was performed on tumor sections from RT2/HRG^+/+^ and RT2/HRG^−/−^ mice. Data are presented as percentage of Ki67 positive cells of total cell numbers. Each dot represents the value from an individual animal (n = 4 for each group). (D–F) Stereological quantification of vascular parameters: length, volumetric and surface densities. Each dot represents the mean value from one individual animal (n = 7 for each group). Statistical analyses were performed with a two-tailed Mann-Whitney test, vertical bars represent standard deviation, * p≤0.05. Scale bars represent 50 µm in A and 100 µm in B.

To investigate why tumors were larger in HRG-deficient mice, we analyzed the proliferative and apoptotic status, as well as the vascularization, of the dissected tumors. Immunohistochemical stainings directed against Ki67 revealed a significantly increased proliferation in tumors from the RT2/HRG^−/−^ group compared to the RT2/HRG^+/+^ group (Fig. 3A and C). No difference in apoptosis was detected as judged from immunohistochemical stainings directed against cleaved caspase-3 (data not shown).

We have in a previous study shown that lack of HRG affects the angiogenic switch in the RIP1-Tag2 model with significantly elevated numbers of angiogenic islets in RT2/HRG^−/−^ mice as compared with RT2/HRG^+/+^ littermates at 7 weeks of age [3]. To investigate how lack of HRG affects pathological angiogenesis in established tumors we performed stereological quantification [21] of tumor vascularization in 12 weeks old RT2/HRG^+/+^ and RT2/HRG^−/−^ mice. This method estimates the total length, volume and surface area of the vasculature per tumor volume. In contrast to our findings regarding the angiogenic switch, we could not detect any statistically significant differences between RT2/HRG^+/+^ and RT2/HRG^−/−^ mice with respect to vascularization of established tumors (Fig. 3B, D–F).

### Enhanced platelet aggregation in HRG-deficient mice

**Figure 4:**
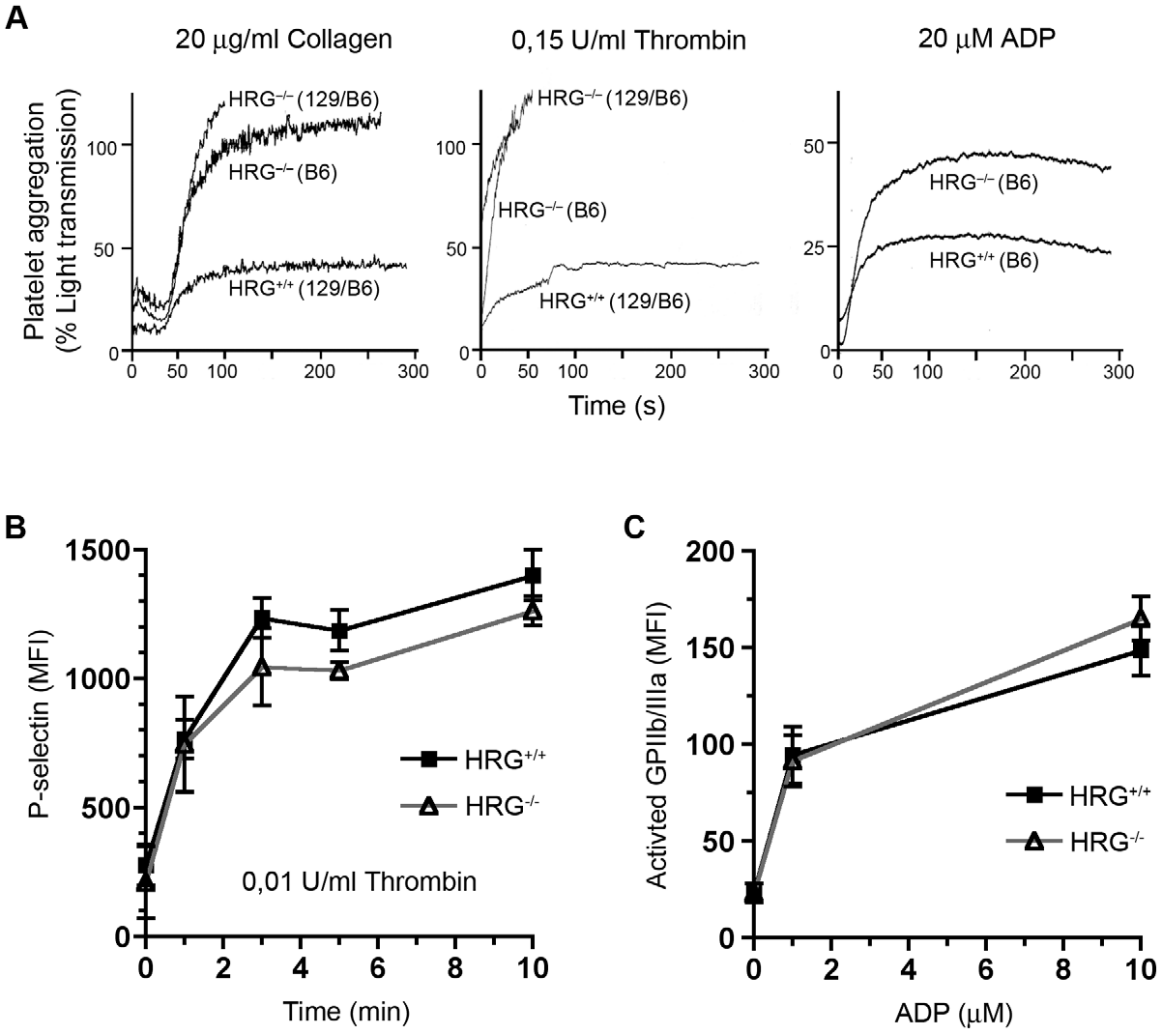
Enhanced aggregation of platelets in HRG^−/−^ mice. (A) Collagen, thrombin and ADP-induced platelet aggregation in PRP from HRG^+/+^ and HRG^−/−^ mice on a pure C57BL/6 (B6) or on a mixed 129/Ola, C57BL/6 (129/B6) genetic background was measured with an aggregometry assay. Aggregation levels are represented by light transmission through PRP over time. (B–C) Flow cytometric analyses of thrombin- and ADP- induced activation of platelets presented by mean fluorescent intensity (MFI). Ligands were added at time = 0. Each symbol (squares = HRG^+/+^, open triangles = HRG^−/−^) represents the mean value from three independent measurements. Vertical bars represent standard deviation. (B) P-selectin after activation with 0,01 U/ml thrombin measured at 0, 1, 3, 5 and 10 minutes. (C) Activated fibrinogen receptor (GPIIb/IIIa) after 15 minutes stimulation with 1 or 10 µM ADP.

HRG^−/−^ mice have a shorter bleeding time than wild type mice [7], suggesting that platelet function may be regulated by HRG. To address this possibility we analyzed whether activation of platelets was affected in HRG-deficient mice. Platelet activation can be measured by their extent of aggregation following activation *in vitro*. For this purpose an aggregometer was used and light transmission recorded over time. As platelets aggregate, light transmission is increased. Platelet-rich plasma (PRP) was prepared from RT2-negative HRG^+/+^ and HRG^−/−^ mice and platelets were activated by the addition of collagen (20 µg/ml), thrombin (0,15 U/ml) or ADP (20 µM). All three platelet activators enhanced aggregation in HRG^−/−^ PRP compared to HRG^+/+^ PRP (Fig. 4A). The HRG deficient mice used for this assay were either on a pure C57BL/6 (B6) or a mixed 129/Ola,C57BL/6 (129/B6) genetic background.

To determine if this fast aggregation was accompanied by an increased expression of platelet surface activation markers, we activated platelets from HRG^+/+^ and HRG^−/−^ mice with thrombin or ADP and analyzed expression of P-selectin and the activated fibrinogen receptor (GPIIb/IIIa) by flow cytometry in whole blood. No difference in the upregulation of these activation markers could be detected between platelets from wild type and HRG-deficient mice (Fig. 4B and C). These data indicate that the enhanced platelet aggregation in mice lacking HRG is most likely caused by an alteration in the plasma milieu surrounding the platelets, rather than related to the activation status of the platelets *per se*.

### The level of circulating platelets is regulated by HRG

**Figure 5:**
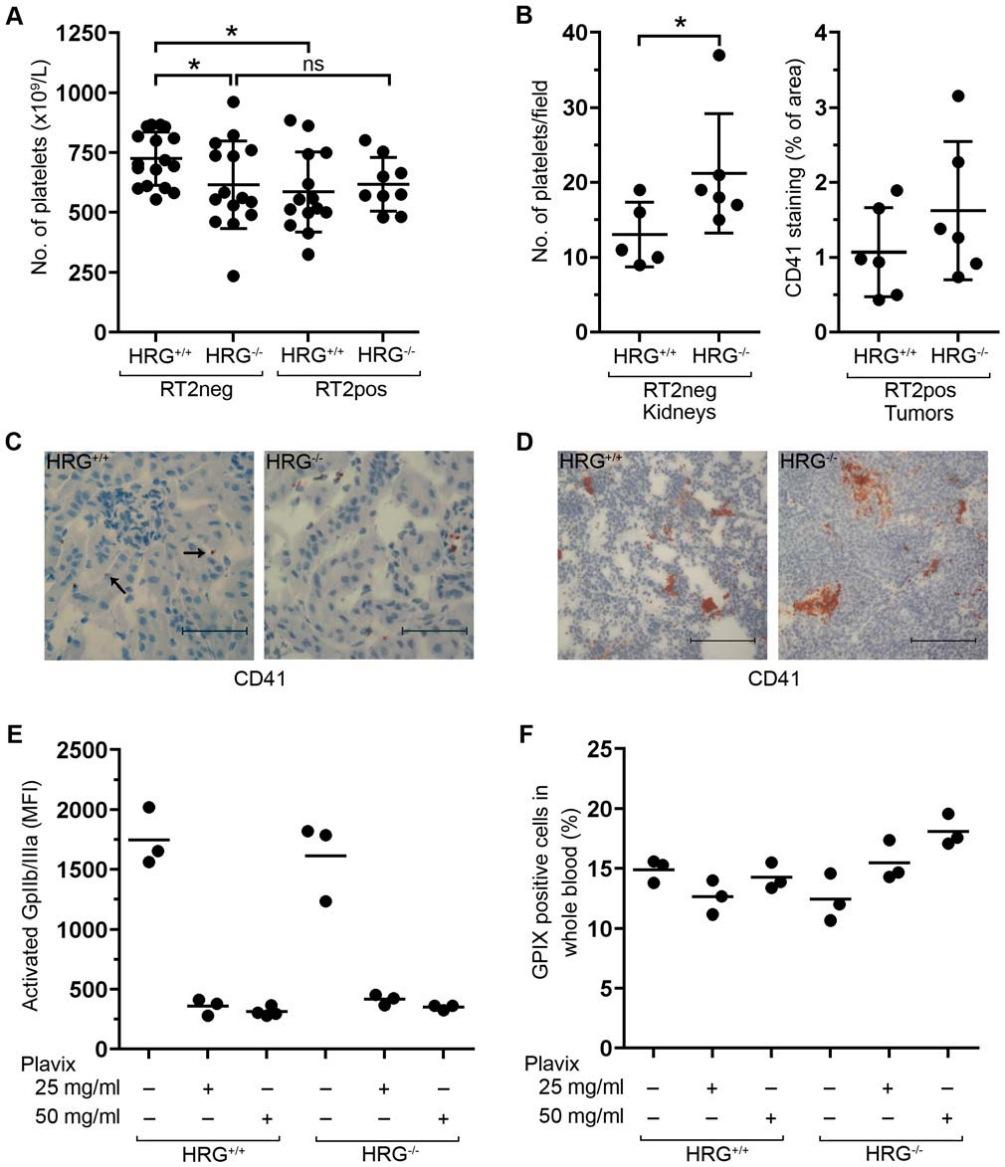
HRG regulates platelet levels in the circulation. (A) Platelet numbers in whole blood from RT2 positive or negative mice with different HRG genotypes (HRG^+/+^ or HRG^−/−^). (B) Left panel; Quantification of the number of platelets/field in kidney tissue from healthy (RT2-negative) HRG^+/+^ or HRG^−/−^ mice. Right panel; Quantification of the area percentage stained positive for the platelet marker CD41 in tumor tissue from RT2-positive HRG^+/+^ and HRG^−/−^ mice. (C, D) Immunohistochemical staining directed against CD41 was performed on kidney from RT2-negative mice (C) and tumor tissue from RT2-positive mice (D) from both genotypes. Arrows indicate platelets. (E) HRG^+/+^ and HRG^−/−^ mice were treated with Plavix (25 mg/ml or 50 mg/ml in drinking water) for three days. Platelet activation in whole blood was measured after stimulation with 10 µM ADP by flow cytometric analysis of the activated fibrinogen receptor (GPIIb/IIIa). (D) Platelet levels after Plavix treatment were measured by flow cytometric analysis in whole blood and are presented as the percentage of GPIX positive cells. Statistical analyses were performed with a two-tailed Mann-Whitney test, vertical bars represent standard deviation, * p≤0.05, ns = non significant. Scale bars represent 50 µm in C and 100 µm in D.

To further analyze platelet activation *in vivo* in HRG knockout mice, we measured the level of circulating platelets as well as the presence of platelets in tissue from HRG^+/+^ and HRG^−/−^ mice. Activated platelets leave the circulation and become arrested in the vascular bed. A reduced number of platelets in the blood can therefore reflect an increase in platelet activation. We analyzed the number of circulating platelets in whole blood from healthy (RT2-negative) wild type and HRG-deficient mice using a cell counter. There was a significant decrease in the number of platelets in blood from HRG^−/−^ mice compared to wild type (Fig. 5A), supporting the conclusion that platelets are more easily activated in the absence of HRG. It is well established that tumors can promote coagulation and platelet activation, for instance by expression of tissue factor [22]. In agreement, platelet levels were significantly reduced in blood from wild type mice with insulinoma (RT2/HRG^+/+^) compared to healthy wild types (HRG^+/+^) (Fig. 5A). However, RT2-positive HRG^−/−^ mice (RT2/HRG^−/−^) showed no further decrease in platelet numbers compared to HRG^−/−^ mice without tumors (HRG^−/−^). Healthy HRG-deficient mice had in fact similar platelet levels as wild type mice with insulinoma (Fig. 5A).

To determine the amount of platelets arrested in tissue in healthy (RT2-negative) mice, immunohistochemical analysis of kidney from wild type and HRG knockout mice was performed using an antibody against the platelet marker CD41. As can be seen in figure 5B (left panel) and C, a significantly higher number of platelets was detected in tissue from mice lacking HRG. When the same analysis was performed on tumor tissue, as well as kidney (data not shown), from RT2-positive HRG^+/+^ and HRG^−/−^ mice, no statistically significant difference with respect to CD41 staining could be detected between the two groups of mice (Fig. 5B (right panel) and D). This result is in agreement with the similar levels of circulating platelets in RT2-positive (HRG^+/+^ and HRG^−/−^) mice, regardless of genotype (Fig. 5A), and probably reflects the platelet activating properties of tumor cells.

To directly address if enhanced platelet activation caused the reduction in circulating platelets in mice lacking HRG, we employed the drug Plavix (clopidogrel), which inhibits platelet activation via the ADP receptor P2Y. Two doses of the inhibitor were administered via the drinking water; 30 mg/kg/day ( = 0,25 mg/ml) or 60 mg/kg/day ( = 0,50 mg/ml) during three days. To assess the efficacy of the treatment, blood was drawn by cardiac puncture and activation of the fibrinogen receptor (GPIIb/IIIa) was analyzed by flow cytometry after ADP activation *in vitro*. Both doses blocked platelet activation by ADP, in wild type as well as in HRG-deficient mice (Fig. 5E). We next analyzed whether treatment with Plavix could affect platelet levels in the circulation of wild type and HRG-deficient mice. Platelet levels were measured by flow cytometric analysis of mouse whole blood using a FITC-conjugated GPIX antibody. HRG-deficient mice treated with 25 and 50 mg/ml Plavix showed a dose-dependent increase in platelet levels in the circulation compared to untreated mice (Fig. 5F). In contrast, Plavix induced no change in platelet levels in wild type mice. These data support the conclusion that platelets are lost from the circulation due to increased activation in mice lacking HRG.

### Platelet depletion attenuates the pro-angiogenic phenotype of HRG-deficient RIP1-Tag2 mice

**Figure 6:**
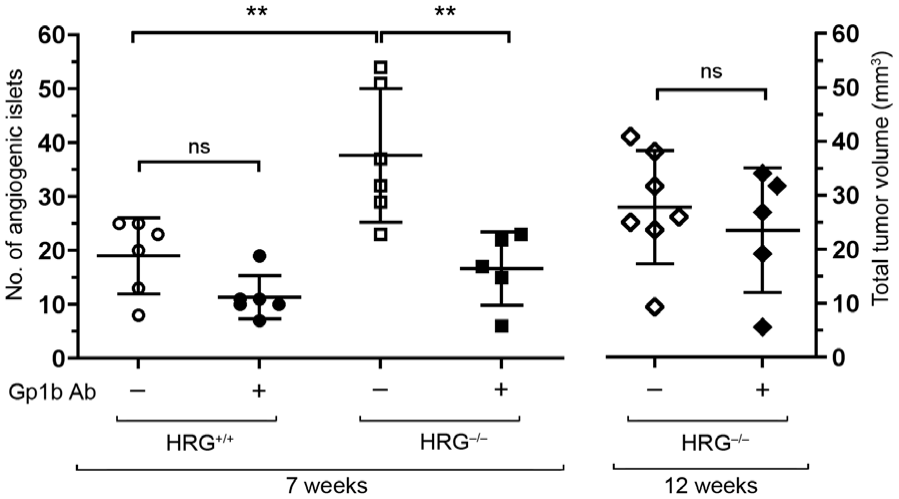
Platelet depletion suppresses the increased angiogenic switch in RT2/HRG^+/+^ mice. RT2/HRG^+/+^ and RT2/HRG^−/−^ mice (n = 6/group) were rendered thrombocytopenic by treatment with an anti-GP1bα antibody during two weeks before the onset of the angiogenic switch. At 7 weeks of age angiogenic islets were counted in each animal. A second group of RT2/HRG^−/−^ mice were treated with the anti-GP1bα antibody during two weeks after the angiogenic switch and tumor volumes analyzed at 12 weeks of age (n = 6/group). (Data on untreated animals at 7 weeks were originally published in [3].) Statistical analyses were performed with a two-tailed Mann-Whitney test, vertical bars represent standard deviation, ** p≤0.001, ns = non significant.

Lack of HRG enhances the angiogenic switch [3] and, as demonstrated here, growth of insulinoma in the RIP1-Tag2 mouse. The angiogenic switch is a prerequisite for the transition of hyperplastic tissue into a growing tumor. To find out whether there is a connection between the enhanced angiogenic switch and the increased platelet activation in HRG-deficient mice, we depleted RT2/HRG^+/+^ and RT2/HRG^−/−^ mice of platelets using an antibody binding to the platelet adhesion molecule GP1bα. The mice were treated during two weeks prior to analysis of angiogenic islets at the age of seven weeks. Platelet depletion significantly reduced the number of angiogenic islets in the RT2/HRG^−/−^ group, but did not affect the number of angiogenic islets in RT2/HRG^+/+^ mice (Fig. 6). When administration of the anti-GP1bα antibody was initiated at a later stage (age nine weeks), after the onset of the angiogenic switch, platelet depletion did not suppress tumor growth in HRG-deficient mice (Fig. 6).

In conclusion, our data suggests that increased platelet activation is the primary cause of the elevated angiogenic switch in HRG-deficient RIP1-Tag2 mice.

## Discussion

In the present study we describe three main findings; 1) tumor growth is significantly increased in HRG-deficient mice, 2) platelet activation is enhanced in mice lacking HRG and 3) the increased platelet activation mediates the accelerated angiogenic switch seen in HRG-deficient mice. These findings are discussed below.

The transgenic RIP1-Tag2 mouse is an orthotopic model of multistage carcinogenesis and believed to better reflect the stepwise process of tumor development via distinctive stages, than conventional subcutaneous models with injected tumor cells [19], [23]. The RIP1-Tag2 mouse is well suited for studies of angiogenesis, since one of the stages during tumor progression is characterized by induction of angiogenesis; the angiogenic switch. We have recently reported that the angiogenic switch is significantly elevated in HRG^−/−^ mice with an approximately two times increase in the number of angiogenic islets [3]. In the present study, HRG^−/−^ mice were found to have a larger tumor volume compared to their HRG^+/+^ littermates. We also noted that HRG^+/−^ mice have an intermediary tumor volume (data not shown) between HRG^+/+^ and HRG^−/−^ mice, which may be related to the fact that mice with one inactivated HRG allele have approximately half the serum concentration of HRG-protein compared to HRG sufficient mice [7]. At 12 weeks of age, HRG^−/−^ mice have an approximately two times larger tumor volume than HRG^+/+^ mice. The correlation - with the same increase in number of angiogenic islets as the increase in tumor volume at 12 weeks – probably reflects that the islets that have undergone the angiogenic switch receive a headstart in the carcinoma development. The reason why we do not see an increased number of tumors is likely because growing islets and/or tumors fuse and become larger tumors. The difference in tumor volume between HRG^+/+^ and HRG^−/−^ mice was enhanced over time with a three times increase in total tumor burden at week 15. This finding is in agreement with the increased proliferation detected in tumor tissue from mice lacking HRG. These data support a role of HRG as an endogenous regulator of tumor growth.

We did not detect any significant difference between HRG^+/+^ and HRG^−/−^ mice with respect to vascularization of established tumors. The reason for this finding is not known but might relate to the particular tumor that is being studied. Established Rip1-Tag2 tumors are highly vascularised in wild type mice and lack of HRG may not be able to promote this further. In contrast, lack of HRG significantly enhances the early onset of pathological angiogenesis in this model [3].

HRG-deficient mice are viable and fertile, but have a coagulation defect resulting in shorter bleeding times [7]. This is an expected phenotype, since HRG has been implicated in regulation of the coagulation system in a number of previous studies [4] [24]. HRG constitutes one of several examples of molecules regulating both angiogenesis and hemostasis. The reason for this dual function is unclear, but may reflect an inherent requirement for strict regulation of the angiogenic process during hemostasis. Angiogenesis must be counteracted at the early stages of vascular injury and platelet adhesion. At a later stage, when the clot has stabilized, angiogenesis is required for tissue regeneration and a new vessel wall is formed from activated endothelial cells. The different steps in this process need to be carefully orchestrated to prevent hemorrhage [8], [13].

Motivated by the reported coagulation defect in HRG-deficient mice, we investigated their platelet activation status and found a significantly faster *in vitro*-aggregation in PRP from HRG-deficient mice compared to wild type using three established platelet-activators: collagen, thrombin and ADP. Furthermore, the level of circulating platelets was decreased in mice lacking HRG, a likely consequence of activation-induced tissue arrest. The reason behind the increased platelet activation in mice that lack HRG is not known, but currently under investigation. Our data indicate that the enhanced platelet aggregation is induced by a factor outside the platelets, since their expression levels of activation markers such as the fibrinogen receptor GPIIb/IIIa and P-selectin were similar in wild type and HRG-deficient mice. It has been reported that the interaction of HRG with fibrinogen delays the conversion to fibrin [25], which could be one explanation for the faster platelet aggregation in HRG^−/−^ mice. However, we observed increased platelet aggregation in HRG-deficient plasma after stimulation with ADP, which most likely does not involve fibrinogen conversion to fibrin.

Platelets have in several different experimental settings been shown to stimulate angiogenesis [11], [14], [15], [16], [17], even though they contain both positive and negative regulators of blood vessel formation [16], [26]. Since HRG-deficient mice display an enhanced coagulation and increased platelet activation, we hypothesized that there could be a connection between this phenotype and our previous finding of an elevated angiogenic switch in these mice [3]. To test this hypothesis we rendered the mice thrombocytopenic by intraperitoneal injections of a GP1bα antibody. This treatment suppressed the increase in number of angiogenic islets in HRG-deficient RIP1-Tag2 mice to wild type level, showing that an enhanced activation of platelets mediates the increased angiogenic switch in these mice. Interestingly, when administration of the anti-GP1bα antibody was initiated after the onset of the angiogenic switch, platelet depletion did not suppress tumor growth in HRG-deficient mice. We therefore conclude that platelets play a crucial role in the early stages of tumor development but are of less significance once angiogenesis has been initiated.

The mechanism behind this contribution of activated platelets to the angiogenic switch is not clear but could involve secretion of proangiogenic factors that stimulate angiogenesis. In addition, platelets contain a number of proteases that upon activation and degranulation of the platelets can increase the bioavailability of matrix-bound growth factors, such as VEGF. Moreover, a fibrin clot may provide a favourable matrix supporting angiogenesis. Another possibility is that other types of blood cells, such as monocytes/macrophages or neutrophils, form complexes with the activated platelets, thereby facilitating their entry into the tumor stroma. Monocytes/macrophages and neutrophils have in several studies been demonstrated to have the capacity to stimulate tumor vascularization and growth [27], [28]. However, preliminary data from our lab indicate that there are no differences between HRG^+/+^ and HRG^−/−^ mice with respect to the number of infiltrating inflammatory cells into the tumor tissue. There could however still be functional differences between these cells in the two genotypes.

We have recently shown that a proteolytic fragment of HRG, corresponding to the antiangiogenic domain, is present in human tissue. In addition, we show that this HRG-fragment can bind to endothelium in the presence of activated platelets and hence exert its antiangiogenic effect [3]. This means that the enhanced angiogenic switch in HRG-deficient mice may have two components; 1) increased platelet activation, due to lack of HRG, that stimulates angiogenesis by the mechanisms mentioned above and 2) the antiangiogenic HRG-fragment that counteracts angiogenesis in the presence of activated platelets is missing. The part of HRG that regulates platelet aggregation may be distinct from the antiangiogenic fragment, which is derived from the His/Pro-rich domain.

In summary, this study shows for the first time that tumor growth is enhanced in mice that lack HRG. Moreover, we show that HRG-deficient mice have increased platelet activation. Finally, we demonstrate that the elevated platelet activation in HRG-deficient mice accelerates the angiogenic switch in the RIP1-Tag2 tumor model. These data further establish platelets as regulators of angiogenesis, especially in a pathological setting. Moreover, we can firmly establish a role for HRG as a modulator of both hemostasis and pathological angiogenesis.
